# Systems level profiling of arginine starvation reveals MYC and ERK adaptive metabolic reprogramming

**DOI:** 10.1038/s41419-020-02899-8

**Published:** 2020-08-20

**Authors:** Caitlyn B. Brashears, Meltem Barlin, William R. Ehrhardt, Richa Rathore, Matthew Schultze, Shin-Chen Tzeng, Brian A. Van Tine, Jason M. Held

**Affiliations:** 1 Department of Medicine, Washington University in Saint Louis School of Medicine, St. Louis, MO 63110 USA; 2 Siteman Cancer Center, Washington University in Saint Louis School of Medicine, St. Louis, MO 63110 USA; 3 Department of Anesthesiology, Washington University in Saint Louis School of Medicine, St. Louis, MO 63110 USA

**Keywords:** Cancer metabolism, Sarcoma, Proteomics

## Abstract

Arginine auxotrophy due to the silencing of argininosuccinate synthetase 1 (ASS1) occurs in many carcinomas and in the majority of sarcomas. Arginine deiminase (ADI-PEG20) therapy exploits this metabolic vulnerability by depleting extracellular arginine, causing arginine starvation. ASS1-negative cells develop resistance to ADI-PEG20 through a metabolic adaptation that includes re-expressing ASS1. As arginine-based multiagent therapies are being developed, further characterization of the changes induced by arginine starvation is needed. In order to develop a systems-level understanding of these changes, activity-based proteomic profiling (ABPP) and phosphoproteomic profiling were performed before and after ADI-PEG20 treatment in ADI-PEG20-sensitive and resistant sarcoma cells. When integrated with metabolomic profiling, this multi-omic analysis reveals that cellular response to arginine starvation is mediated by adaptive ERK signaling and activation of the Myc–Max transcriptional network. Concomitantly, these data elucidate proteomic changes that facilitate oxaloacetate production by enhancing glutamine and pyruvate anaplerosis and altering lipid metabolism to recycle citrate for oxidative glutaminolysis. Based on the complexity of metabolic and cellular signaling interactions, these multi-omic approaches could provide valuable tools for evaluating response to metabolically targeted therapies.

## Introduction

The silencing of argininosuccinate synthetase 1 (ASS1) expression disrupts the urea cycle in many types of cancer^1–4^. Importantly, loss of ASS1 expression renders cancer cells dependent on extracellular arginine, as de novo arginine synthesis is reliant on ASS1^4^. While the adaptive function of ASS1 silencing is not yet fully understood, current data suggest that it is beneficial for the production of biomass^5,6^. Cancers that silence ASS1 have been shown to have a more aggressive clinical course, as silencing is associated with poorer overall survival and metastasis-free survival in numerous subtypes of cancer^7–10^.

To exploit this metabolic deficiency, multiple arginine destruction enzymes have been developed, including arginase, arginine decarboxylase, and arginine deiminase^1,11^. The most clinically relevant is PEGylated arginine deiminase (ADI-PEG20), which is currently in clinical trials^12^. ADI-PEG20 converts extracellular arginine to citrulline, which cannot be metabolized into arginine in the absence of ASS1^13–15^. Early development of ADI-PEG20 as a monoagent failed to demonstrate a survival advantage, likely due to the rapid re-expression of ASS1 in tumors^16^. Due to the high adaptability of tumor metabolism, most metabolically active drugs are not effective when used as monoagents^15^. However, investigations of the metabolic reprogramming that ASS1-negative tumors undergo as they re-express ASS1 have revealed additional vulnerabilities in ADI-PEG20 sensitive sarcomas^7,8,15,17^. To date, many of these studies have relied upon metabolomic and genetic stratigies to understand the development of resistance to ADI-PEG20 treatment in ASS1 negative tumors. However, given the complex nature of cell signaling and cellular metabolism, other “omics” techniques, such as proteomics, may provide additional insight into threapeutically actionable targets.

Proteomic profiling can assess multiple potential aspects of protein regulation, such as protein abundance or protein post-translational modifications^18–20^. Alternatively, activity-based proteomic profiling (ABPP) can evaluate changes in protein activity^21^, kinase activity^22^, or ligand binding events^23^. Changes in protein expression or protein-protein interactions invariably contribute to ABPP as well^24–26^. Ultimately, ABPP integrates multiple informative proteomic parameters and provides a broad view of proteomic regulation. For example, ABPP can identify adaptive kinomic changes based on either altered kinase expression or activity^22^.

The mechanisms of developing resistance to arginine starvation in sarcomas have been partially defined, and include stabilization of nuclear Myc^17^, and increased glutamine anaplerosis in order to produce aspartate^15^. In addition, others have examined mechanisms of ASS1 re-expression^27,28^ and Deptor regulation^29^. However, the underlying proteomic changes that initiate these events and coordinate metabolic reprogramming remain unknown. We pursued systems biology profiling to understand resistance to arginine starvation, as these approaches have proven effective in delineating the adaptive changes involved in highly pleiotropic phenotypes such as drug resistance^30,31^, Myc activation, and various metabolic changes^32,33^.

To understand ADI-PEG20-resistance of ASS1-negative sarcomas at a systems level, we performed multi-omic profiling using phosphoproteomics and activity-based proteomics, and coupled these data with existing metabolomic analyses^15^. ADI-PEG20-senstive leiomyosarcoma cells (SKLMS-1) have a much more dynamic phosphoproteomic response to ADI-PEG20 than a resistant angiosarcoma cell line (PCB-011). This includes increased phosphorylation of PDHA (pyruvate dehydrogenase) at Ser^293^, that inhibits entry of pyruvate into the mitochondrial TCA cycle via decarboxylation^15^. ABPP profiling reveals that glutamine anaplerosis is facilitated by proteomic changes that drive the production of OAA (oxaloacetate) by glutamine and by anaplerotic carboxylation of pyruvate, as well as the inhibition of lipid metabolism to recycle citrate to the TCA cycle. In addition, ABPP profiling reveals a Myc–Max transcriptional network that is regulated by adaptive changes in MAPK1 and MAPK2 upon ADI-PEG20 treatment in SKLMS-1 cells. Therefore, we have demonstrated that multi-omic profiling can be utilized to delineate systems-level regulatory signaling networks mediating drug sensitivity and resistance. Due to the complex nature of metabolic and cell signaling interactions, these approaches could provide valuable tools for evaluating resistance and escape to metabolically targeted cancer therapies.

## Materials and methods

### Materials

All materials were from Sigma unless otherwise noted. ActivX desthiobiotin ATP kinase enrichment kit and BCA assay were from Pierce. Mass spec grade trypsin was from Promega. Amicon Ultra Centrifugal Filters and C18 ziptips were from Millipore. Bondbreaker TCEP, 5 mL 7 K MWCO Zeba spin desalting columns, and formic acid were from ThermoFisher. Oasis HLB 1 cc extraction columns were from Waters. Lysis buffer for phosphorylation analysis was from Cell Signaling Technologies. Sequencing grade trypsin was from Promega. Ni-NTA agarose beads were from Qiagen. MEM (11095-072) and Penicillin-Streptomycin (15140122) for cell culture were from ThermoFisher Scientific. Fetal Bovin Serum for cell culture was obtained from R&D Systems (S111560). The antibodies used in the immunoassays are as follows: ASS1(Polaris), ERK1/2 (CST 4695), phospho-ERK1/2 (Thr202/Tyr204) (CST 4370), cMyc (Abcam ab11917), phospho-cMyc (S62) (abcam ab51156).

### Cell culture

SKLMS-1 was acquired from ATCC. PCB-011 was generously provided by Dr. Charles Keller (Children’s Cancer Therapy Development Institute). SKLMS-1 and PCB-011 were cultured at 37 °C in 5% CO_2_ in Minimum Essential Media (MEM) supplemented with 10% fetal bovine serum (FBS), penicillin–streptomycin (1:1000), and plasmosin (InvivoGen ant-app). Cells were confirmed to be mycoplasma negative with the mycoalert kit (Lonza LT07-418). SKLMS-1 LTAT cell lines were generated as previously described^15^. NucRed Cell lines were generated with IncuCyte NucLight Red Lentivirus Reagent (EF-1 Alpha Promoter, Puromycin selection, cat. No. 4476) according to manufactor protocol.

### Proliferation and cell death assays

For analysis of cellular response to treatment with ADI-PEG20, SKLMS-1 and PCB-011 were seeded at 2500 cells per well in a 96-well plate one day prior to the assay. Phenol red free media containing 10% FBS and 2 mM glutamine was pretreated with 1 µg/mL of ADI-PEG20 on day prior to the assay. On the day of the assay, the media was exchanged for ADI-PEG20 pretreated media or phenol free media in the untreated control.

For evaluation of SKLMS-1 response to inhibition of ERK and cMyc signaling pathways NucRed SKLMS-1 WT and LTAT cell lines were utilized. In brief, SKLMS-1 WT and LTAT cell lines were seeded at 5000 cells/well in a 96-well plate 1 day prior to treatment. Phenol red free media containing 10% FBS and 2 mM glutamine was pretreated with 1 µg/mL of ADI-PEG20 for 24 h. On the day of the assay the media was exchanged with media containing 50 nM YOYO-1 Iodide (ThermoFisher Y3601) and: untreated phenol free media, ADI pretreated phenol free media, 1.6 µM Trametinib (LC Laboratories T-8123), and/or 5 µM 10058-F4 (Selleckchem S7153). Cell proliferation was measured using NucRed nuclear counts. Cell Death was measured using YOYO-1 Iodide counts. Percent Cell death was calculated by normalizing YOYO-1 Iodide counts to the total number of cells in the well. All images were collected using the IncuCyte Live Cell Imaging System and data was analyzed using IncuCyte S3 imaging software (Sartorius Ann Arbor, MI).

### Immunoassays

For analysis of protein expression cells were seeded at 200,000 cells per well in a 10 cm dish and MEM was pretreated with ADI-PEG20 (1 µg/mL) for 24 h. On the day of the assay the media was exchanged for ADI-PEG20 pretreated media or fresh MEM in the untreated control. After 72 h of treatment cells were lysed with 1× cell lysis buffer (9803, Cell Signaling Technology). Lysates were run on a ProteinSimple Wes automated western blot using the instrument default settings and the ProteinSimple standard protocol. Protein Simple Compass was utilized for the data analysis.

### ABPP using ATP resin

Cells in a 10 cm dish were lysed and assayed using the Pierce kinase enrichment kit with the ActivX desthiobiotin-ATP probe per manufacturer’s instructions and prepared for liquid chromatography–mass spectrometry (LC–MS). Three independent biological replicates were performed. Briefly, cells were treated with ADI-PEG20 for 72 h, trypsinized and pelleted at 1000×*g* for 5 min. The pellet was washed once with 5 mL PBS and lysed in 1 mL Pierce IP lysis buffer with the included protease/phosphatase inhibitors added. Lysates were desalted with 5 mL 7 K MWCO Zeba according to manufacturer’s instructions, diluted to 2 mg/mL in lysis buffer, and labeled with 20 µM desthiobiotin-ATP for 10 min at room temperature. Proteins were enriched with the IP lysis buffer plus 8 M urea with 50 µL 50% slurry of the high capacity streptavidin sepharose included in the kit, rotating end-over-end for 1 h. Resin was washed 3 times with lysis buffer supplemented with 4 M urea prior to elution with 500 µL 0.5% sodium dodecyl sulfate, 1% B-mercaptoethanol in 0.1 M Tris pH 6.8 and heated at 95 °C for 5 min. A second, identical elution was performed and combined with the first. Lysates were reduced (10 mM DTT, 25 min, 60 °C), alkylated with iodoacetamide (18 mM, 30 min) and concentrated with Millipore Amicon Ultra spin columns (UltraCel, 10 K MWCO). Proteins were precipitated with 5 volumes MeOH:chloroform (4:1, v/v). The interphase was isolated, washed with MeOH, and proteolyzed with 1.25 µg trypsin in 78 µL of 2% acetonitrile overnight at 37 °C at 900 rpm. Samples were acidified with 0.5% formic acid, desalted with C18 zip tips (0.6 µL resin), eluted with 80% acetonitrile, 0.5% formic acid prior to vacuum concentrated to near dryness prior to LC–MS analysis.

### IMAC enrichment of phosphopeptides

Three independent biological replicates were performed. Cells in 10 cm dishes were treated with or without ADI-PEG20 (10 µM) for 72 h, washed with PBS and lysed in 1× Cell Signaling Cell Lysis Buffer plus 1 mM phenylmethylsulfonyl fluoride. Lysates were sonicated in a water bath on ice for 15 s and insoluble material was removed with a 14,000×*g* centrifugation for 10 min at 4 °C. Five 200 µg aliquots of lysate were made for each sample. Each aliquot was desalted with 600 µL GE 2-D Clean-Up kit and processed through to trypsin digest as in ABPP. Lysates were desalted using Oasis HLB columns per manufacturer’s instructions. Samples were then diluted with 1 mL 90% ACN (acetonitrile), and phosphopeptides were enriched with 20 µL of Qiagen Ni-NTA slurry for 30 min at 25 °C with end-over-end rotation. Beads were washed four times with 1 mL 80% ACN, 0.1% trifluoroacetic acid and eluted with 250 µL 50% ACN, 2.5% ammonia, and 2 mM phosphate buffer pH 10. Lysates were acidified to pH <3 with formic acid, vacuum concentrated to dryness, desalted with a C18 ziptip using manufacturer’s instructions, vacuum concentrated to dryness, resuspended in 0.5% formic acid and analyzed by LC–MS.

### Liquid chromatography–mass spectrometry

Samples were analyzed by reverse-phase liquid chromatography–electrospray ionization–MS/MS using an Eksigent cHiPLC Nanoflex microchip system connected to a quadrupole time-of-flight TripleTOF 5600 mass spectrometer (ABSCIEX). The Nanoflex system uses replaceable microfluidic traps and columns packed with ChromXP C18 (200 μm ID × 15 cm, 3 μm particle, 120 Å) for online trapping, desalting, and analytical separations. Solvents composed of water/acetonitrile/formic acid (A, 100/0/0.1%; B, 0/100/0.1%). A 200 ng to 1 µg portion of sample was loaded (typically, 2–10 μl of sample was injected) into column with 98% mobile phase A. After online trapping, peptide mixtures were eluted into analytical column at a flow rate of 800 nL/min using the following gradient: (1) starting at 2% solvent B; (2) 2–5% solvent B from 0 to 12 min; (3) 5–22% solvent B from 12 to 120 min; (4) 22–30% solvent B from 120 to 150 min; (5) 30–80% solvent from 150 to 165 min; and finally 80% (vol/vol) solvent from 165 to 169 min with a total run time of 180 min including mobile phase equilibration. Column was maintained at 35 °C during the run.

Two different mass spectrometric acquisition workflows were performed in this study: (1) *Data dependent acquisitions (DDA):* Mass spectra and tandem mass spectra were recorded in positive-ion and high-sensitivity mode. The nanospray needle voltage was typically 3800 V. After acquisition of each sample, TOF MS spectra and TOF MS/MS spectra were automatically calibrated during dynamic LC–MS and MS/MS auto calibration acquisitions by injecting 50 fmol β-galactosidase. For collision-induced dissociation tandem MS (CID–MS/MS), the mass window for precursor ion selection of the quadrupole mass analyzer was set to ±1 *m/z*. The precursor ions were fragmented in a collision cell using nitrogen as the collision gas. Advanced information-dependent acquisition was used for MS/MS collection on the TripleTOF 5600 to obtain MS/MS spectra for the 20 most abundant parent ions following each survey MS1 scan (allowing typically for 80 ms acquisition time per each MS/MS). Dynamic exclusion features were set to an exclusion mass width of 50 mDa and an exclusion duration of 30 s. (2) *Data independent MS2 acquisitions (DIA):* In the “SWATH” DIA MS2 acquisition, instead of the Q1 quadrupole transmitting a narrow mass range through to the collision cell, a wider window of ~10 *m/z* is passed in incremental steps over the full mass range (*m/z* 400–1250 with 85 SWATH segments, 63 ms accumulation time each, yielding a cycle time of 5.5 s which includes one MS1 scan with 50 ms accumulation time). SWATH MS2 produces complex MS/MS spectra that are a composite of all the analytes within each selected Q1 *m/z* window. The RAW and processed data associated with this manuscript have been deposited to the ProteomeXchange repository with the identifier PXD017043.

### Protein identification and MS1 quantification with MaxQuant

Mass spectral data sets were analyzed and searched with MaxQuant (ver.1.5.2)^34^ against the Uniprot Human Reference Proteome. The MS/MS spectra were deisotoped and filtered such that only the ten most abundant fragments per 100-*m/z* range were retained. The MS/MS spectra were searched with fixed modification of Carbamidomethyl-Cysteine, variable modifications of oxidation (M), acetylation (protein N-term), Gln- > pyro-Glu, and phosphoryation (STY). Search parameters were set to an initial precursor ion tolerance of 0.07 Da, MS/MS tolerance at 40 ppm and requiring strict tryptic specificity with a maximum of two missed cleavages. The minimum required peptide length was set to seven amino acids. Identification false-discovery rate (FDR) was set at 1%. Label-free protein and peptide quantification was performed in MaxQuant and data normalization was done in Perseus. Peptides that were unique in gene level were summed to represent protein expression. Ratios of protein expression in drug treated sample against non-treated sample were calculated. The RAW and processed data associated with this paper have been deposited to the ProteomeXchange repository with the identifier PXD017043.

### Gene set enrichment analysis (GSEA)

GSEA^35^ was performed using GSEA version 2.2.2 from the Broad Institute at MIT. Parameters used for the analysis were as follows. Datasets with protein expression fold changes due to drug treatment were testing for enrichment against BioCarta, Hallmark, Reactome and KEGG gene sets. Number of permutations was set to 1000 to calculate *p*-value and permutation type was set to gene_set. All basic and advanced fields were set to default. Phosphopeptides were assigned to specific genes based on the MaxQuant annotation.

### Skyline data analysis

Skyline software (https://skyline.ms/project/home/begin.view?) was used to manually examine and quantify DIA data. Spectral libraries were generated in Skyline using the DDA database searches of the raw data files. Raw files were directly imported into Skyline in their native file format and only cysteine containing peptides were quantified.

### Gene set enrichment analysis (GSEA)

GSEA^35^ was performed using GSEA version 2.2.2 from the Broad Institute at MIT. Refer to supplemental methods for detailed parameters.

### Kinome tree plot

KinMap^36^ was used to generate the kinome tree based on relative expression in the ABPP dataset.

### X2K

X2K^37^ was performed using default parameters and Networkin as the kinome database. Input was all proteins upregulated by ADI-PEG20 (nominal *p* value ≤ 0.05) in SKLMS-1 cells in the ABPP dataset.

### Statistics

All *t* tests were two sided.

## Results

### Proliferative and morphologic changes of PCB-011 and SKLMS-1 with ADI-PEG20 treatment

**Fig. 1:**
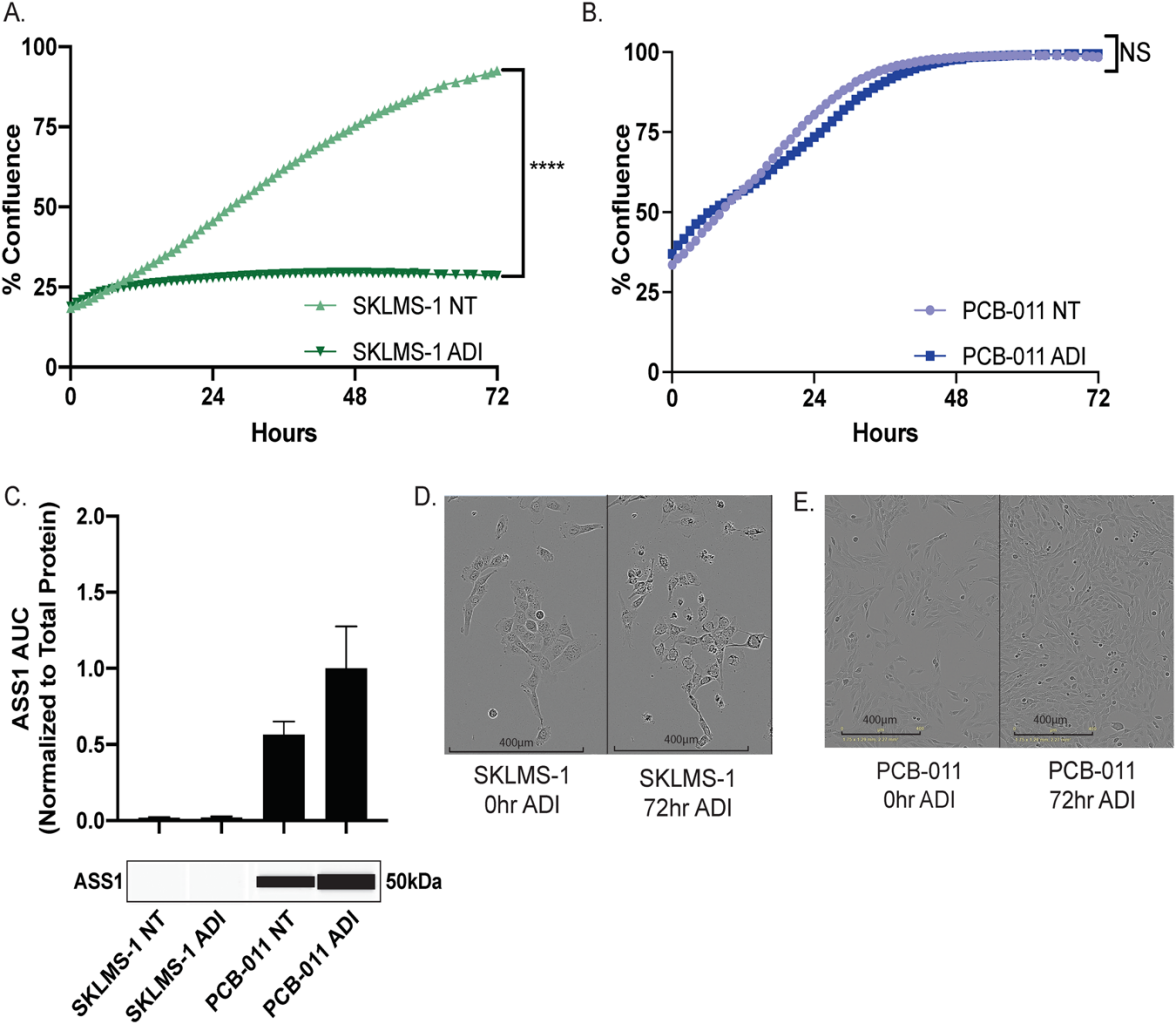
Proliferative and morphologic changes of PCB-011 and SKLMS-1 with ADI-PEG20 treatment. **a** In vitro cell proliferation response to extracellular arginine deprivation with ADI-PEG20 in SKLMS-1. Cell proliferation was measured using cell confluence on the IncuCyte Live Cell Analysis System. (*n* = 3) ****p < 0.0001. **b** In vitro cell proliferation response to extracellular arginine deprivation with ADI-PEG20 in PCB-011. Cell proliferation was measured using cell confluence on the IncuCyte Live Cell Analysis System. (*n* = 3) NS *p* > 0.05. **c** Protein expression of ASS1 compared in untreated and ADI-treated SKLMS-1 and PCB-011 at 72 h. Cell lysates were analyzed with SimpleProtein Wes automated capillary western system. Band density differences were plotted as ASS1 area under the curve normalized to total protein in the capillary (representative *N* = 3); data are represented as mean + SD. **d** Representative (*N* = 3) DIC images of SKLMS-1 at 0 and 72 h of ADI-PEG20 treatment. Images were collected on the IncuCyte Live Cell Analysis System at 20× magnification. **e** Representative (*N* = 3) DIC images of PCB-011 at 0 and 24 h of ADI-PEG20 treatment. Images were collected on the IncuCyte Live Cell Analysis System at 10× magnification.

To systemically identify regulatory networks underlying resistance to ADI-PEG20 we examined two sarcoma cell lines, SKLMS-1 (leiomyosarcoma) and PCB-011 (angiosarcoma). Treatment of SKLMS-1 cells with ADI-PEG20 resulted in cessation of cellular proliferation, indicating that SKLMS-1 cells are sensitive to arginine starvation induced by ADI-PEG20 (Fig. 1a). In comparison, PCB-011 cells were rapidly resistant to ADI-PEG20 as evidenced by their continued proliferation over 72 h of treatment (Fig. 1b). These data are consistent with the ASS1 expression in each cell line, as SKLMS-1 is ASS1-negative, while PCB-011 is ASS1-positive (Fig. 1c). After 72 h of treatment with ADI-PEG-20, PCB-011 increases expression of ASS1, suggesting a rapid adaptation to treatment. Comparatively, SKLMS-1 fails to significantly increase expression of ASS1 after 72 h. Finally, unlike PCB-011, there is a morphological change identified in the SKLMS-1 cell line, as it becomes more spindle-like in response to arginine starvation (Fig. 1d, e). Cumulatively these data demonstrate the SKLMS-1 is sensitive to arginine depletion with ADI-PEG20, while PCB-011 is not responsive.

### Phosphoprotemomic changes as a result of ADI-PEG20 treatment in sensitive and resistant cell lines

Glutamine and glucose metabolic tracing has previously shown that SKLMS-1 cells increase anaplerotic oxidative glutaminolysis to produce aspartate from oxaloacetate in response to ADI-PEG20-induced arginine starvation^15^. In addition, western blots of candidate metabolic regulatory proteins have shown decreased phosphorylation of PKM2 Y^105^ (pyruvate kinase) and LDHA Y^10^ (lactate dehydrogenase), and increased phosphorylation of PDH1 S^300^ in response to ADI-PEG20^15^. In order to gain insight into how proteomic adaptations in ADI-PEG20 sensitive cells promote altered metabolism and cell signaling to survive arginine deprivation, we performed ABPP using an ATP-resin^24^ as well as phosphoproteomic profiling of SKLMS-1 and PCB-011 cells with and without ADI-PEG20 treatment for 72 h. In ADI-PEG20-sensitive SKLMS-1 cells and ADI-PEG20-resistant PCB-011 cells, changes between the untreated condition and 72 h of ADI-PEG20 treatment were compared. PCB-011, a cell line with intrinsic ADI-PEG20 resistance, was utilized as a negative control in the proteomic analysis.

**Fig. 2:**
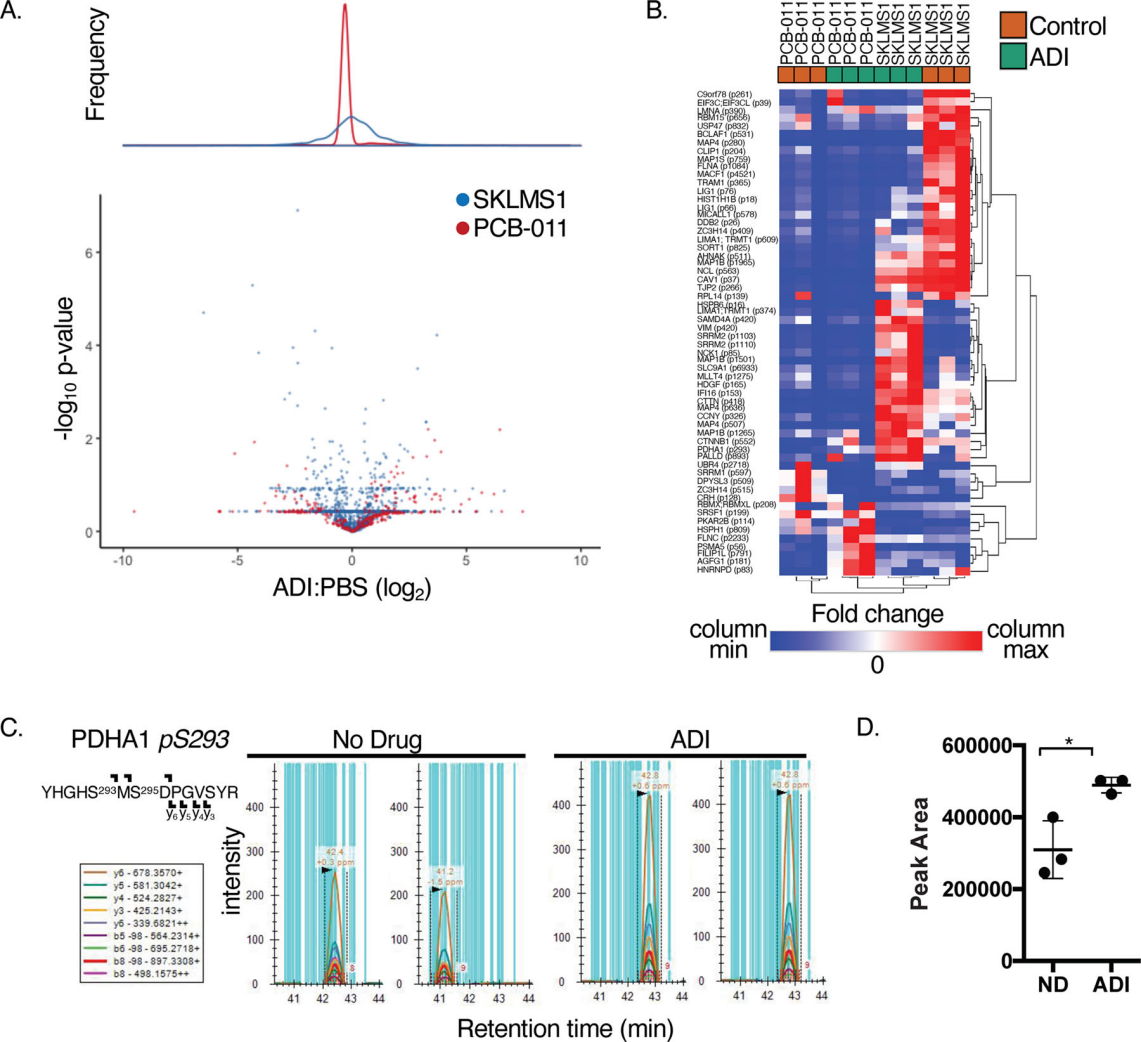
ADI-sensitive SKLMS-1 cells have a dynamic phosphoproteomic response to arginine starvation that regulates pyruvate dehydrogenase and proteins involved in cell morphology and contacts. **a** Phosphoproteomic profiling of SKLMS-1 and PCB-011 sarcoma cells upon ADI-PEG20-PEG (ADI-PEG20) treatment (72 h, *N* = 3 biological replicates). **b** Heatmap of proteins with significantly altered phosphorylation upon ADI-PEG20 treatment. Colors are assigned according to the directionality of deviation from no change (red, up; blue, down). Three biological replicates were analyzed for each cell per conditions. **c** DIA-MS results for phosphorylation of S29 in PDHA1. **d** Quantification of S29 phosphorylation in PDHA1 upon ADI treatment. Two-sided *t* test, *p < 0.05.

We first examined how the phosphoproteome of each cell line responded to ADI-PEG20 treatment. 2551 phosphopeptides were detected with a 1% FDR. Notably, the phosphoproteomic response of ADI-PEG20-sensitive SKLMS-1 cells was much more dynamic than PCB-011 cells, with more phosphopeptides upregulated and downregulated by ADI-PEG20 (Fig. 2a). A heatmap of phosphopeptides altered by ADI-PEG20 treatment also indicates that the SKLMS-1 phosphoproteome is more dynamic than ADI-PEG20-resistant PCB-011 cells when starved of arginine (Fig. 2b).

The phosphorylation of several notable proteins is uniquely upregulated in ADI-PEG20-sensitive SKLMS-1 cells. This includes phosphorylation of PDHA1 S^293^, which inhibits pyruvate entry into the TCA cycle through oxidative decarboxylation^15^ and increases anaplerotic production of oxaloacetate (OAA)^15^. These data are consistent with previous metabolomic analyses^15^. The increase in PDHA1 S^293^ phosphorylation was verified by DIA-MS (data-independent acquisition mass spectrometry)^38^, which was able to clearly distinguish phosphorylation of S^293^ from other serines in the peptide (Fig. 2c). DIA-MS shows a significant (1.6-fold) increase in the expression of pS^293^ upon ADI-PEG20 treatment (Fig. 2d), paralleling the increase in PDHA S^300^ phosphorylation upon ADI-PEG20 treatment^15^. Other phosphoproteins uniquely regulated by ADI-PEG20 treatment in ADI-PEG20-sensitive SKLMS-1 cells include β-catenin, as well as the cell morphology and contact proteins LIMA1, VIM, MAP1B, MLLT4, CTTN, and PALLD, which is consistent with the change to a more fusiform morphology, as noted in ADI-treated SKLMS-1 cells (Fig. 1d).

### SKLMS-1 cells upregulate MAPK signaling and TCA proteins in response to ADI-PEG20, but downregulate lipid metabolism

**Fig. 3:**
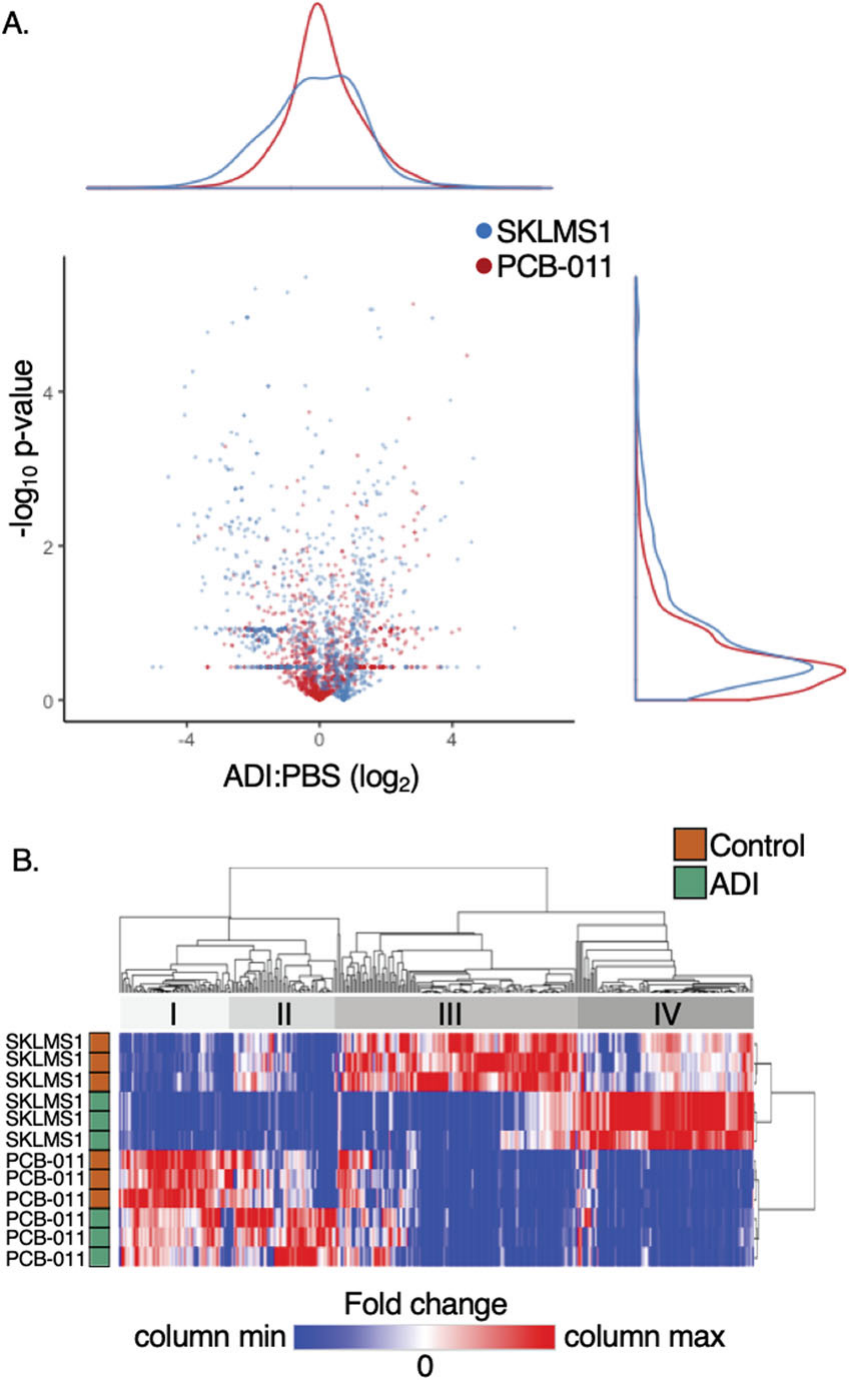
Activity-based proteomic profiling reveals regulation of metabolism and kinases that are unique to ADI-sensitive SKLMS-1 cells upon arginine starvation. **a** ABPP profiling of SKLMS-1 and PCB-011 sarcoma cells upon ADI-PEG20-PEG (ADI-PEG20) treatment (72 h, *N* = 3 biological replicates). **b** Heatmap of proteins with significantly altered phosphorylation upon ADI-PEG20 treatment. Color choices are assigned according to the directionality of deviation from no change (red, up; blue, down). Three biological replicates were analyzed for each cell per conditions.

ABPP detected 1912 proteins, and substantial activity-based proteomic changes in both cell lines upon ADI-PEG20 treatment (Fig. 3a). A clustered heatmap including all proteins that were differentially regulated (*p* value ≤ 0.05) reveals four distinct clusters, indicating that the proteomic response to ADI-PEG20 in each cell line is highly individualized (Fig. 3b).

**Fig. 4:**
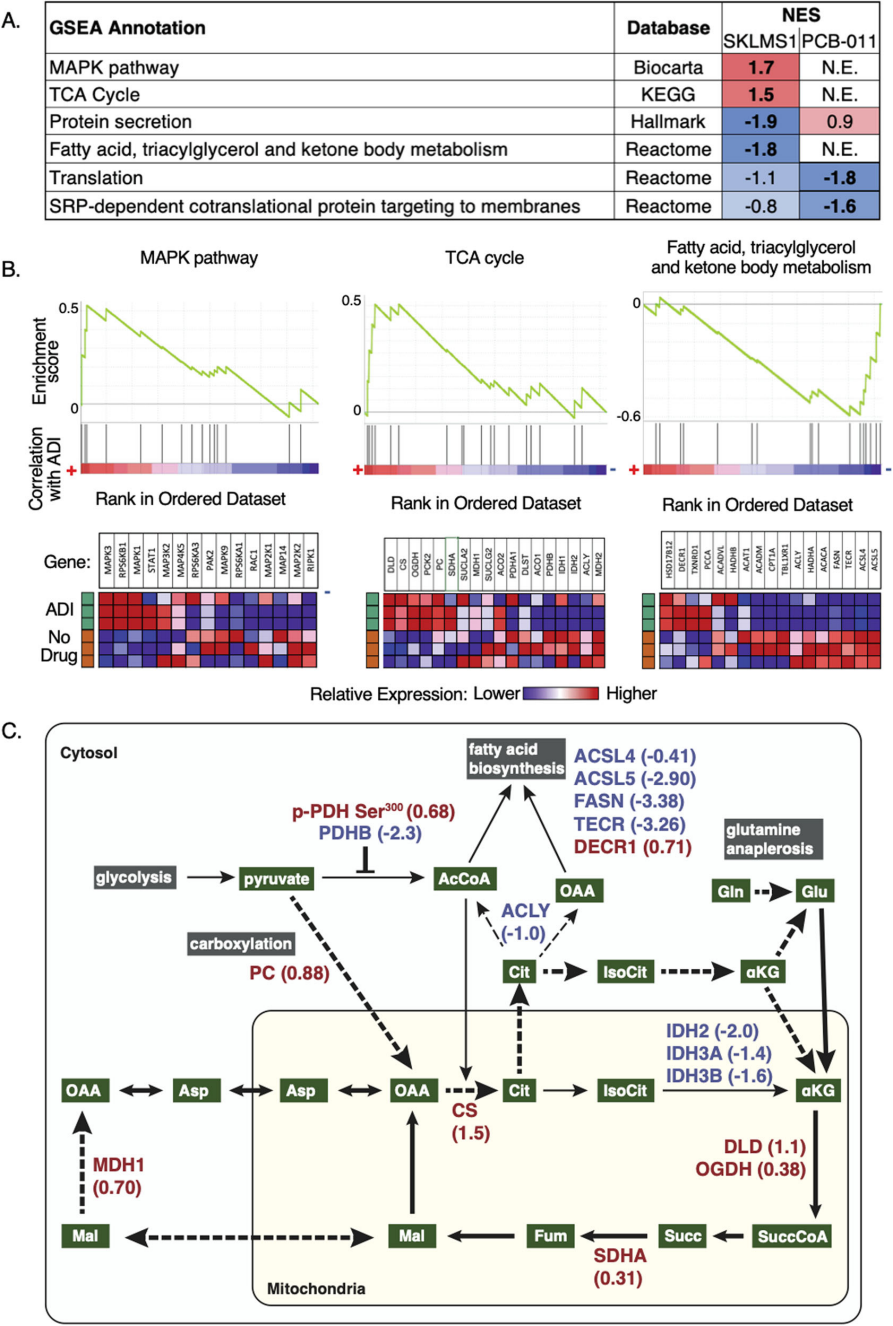
Arginine starvation induces MAPK signaling and coordinated proteomic alterations that promote glutamine anaplerosis, oxaloacetate formation, and inhibit lipid metabolism in SKLMS-1 cells. **a** Normalized enrichment scores (NES) of Annotations enriched from gene set enrichment analysis (GSEA) analysis for ABPP profiling of SKLMS-1 and PCB-011 cells in response to ADI-PEG20 treatment. N.E. indicates Not Enriched and bold font indicates a false-discovery rate < 25%. **b** Relative expression of individual genes in Annotations enriched in SKLMS-1 cells. **c** Schema of glycolysis, TCA cycle and lipid metabolism with ABPP and phosphoproteomic results (log_2_ fold change of ADI-treated compared to untreated, colors based on directionality of deviation from no change (red, up; blue, down)) in SKLMS-1 cells, overlaid with known metabolic changes in SKLMS-1 cells upon ADI treatment^15^ (thick solid lines). Dashed lines indicate altered metabolism proposed by ABPP results. Colors are assigned according to the directionality of deviation from no change (red, up; blue, down).

In order to determine the biological pathways and functions of proteins regulated by ADI-PEG20 treatment, we performed gene set enrichment analysis (GSEA)^35^. GSEA utilizes the fold changes of all proteins detected by ABPP, a more appropriate approach than filtering data by an arbitrary cutoff^39^. MAPK pathway and TCA cycle annotations were enriched in SKLMS-1 cells after ADI-PEG20 treatment but were not enriched in PCB-011 cells (Fig. 4a). In addition, fatty acid, triacylglycerol, and ketone body metabolism was negatively regulated in SKLMS-1 but not in PCB-011 cells (Fig. 4a). PCB-011 cells did not show strong positive enrichment of any pathways but showed negative regulation of translation and SRP-dependent co-translational protein targeting to membranes, which were also observed, albeit less strongly, in SKLMS-1 cells (Fig. 4a). Enrichment plots and heatmaps of expression in SKLMS-1 cells demonstrate coordinated regulation of proteins within the MAPK, TCA cycle, and fatty acid, triacylglycerol, and ketone body metabolism pathways upon ADI-PEG20 treatment (Fig. 4b). Metabolic reprogramming in the TCA cycle has been previously observed in SKLMS-1 cells upon ADI-PEG20 treatment^15^. In addition, regulation of MAPKs and lipid metabolism has been observed in ADI-PEG20-sensitive melanoma^15,40^. These results demonstrate that ADI-PEG20-sensitive SKLMS-1 cells undergo metabolic and kinomic adaptation upon arginine starvation, which is consistent with the existing literature^15,40,41^.

### Arginine starvation induces coordinated proteomic alterations that promote glutamine anaplerosis, oxaloacetate formation, and inhibit lipid metabolism in SKLMS-1 cells

ADI-PEG20 treatment increases glutamine anaplerosis through the TCA cycle, forming oxaloacetate to produce aspartate^15^. However, the specific proteomic alterations that facilitate this metabolic rewiring remain unknown. In order to provide a more complete understanding of the mechanisms underlying the adaptive metabolic rewiring in response to arginine starvation, we focused on the ABPP regulation of individual proteins in the differentially regulated TCA cycle and fatty acid, triacylglycerol, and ketone body metabolism annotations in SKLMS-1 cells upon arginine starvation (Fig. 4b).

Multiple proteomic alterations support enhanced glutamine anaplerosis and utilization of oxaloacetate (OAA). First, multiple enzymes that drive glutamine anaplerosis to OAA were upregulated, including DLD (dihydrolipoamide dehydrogenase), OGDH (oxoglutarate dehydrogenase), and SDHA (succinate dehydrogenase A) (Fig. 4c). In addition, IDH2 (isocitrate dehydrogenase), IDH3A, and IDH3B are substantially downregulated, blocking the reverse activity of the TCA cycle that can occur in cancer^15^, and further directing αKG (alphaketoglutarate) toward OAA. Second, PDHB levels are decreased and pyruvate carboxylase (PC) levels are increased. Together with the increased phosphorylation of PDH S^300^ (Fig. 2d), which inhibits PDH activity^15^, these findings suggest that more pyruvate is directly converted to OAA via anaplerotic carboxylation upon arginine deprivation (Fig. 4c). Third, ABPP finds that while citrate synthase (CS) is upregulated, metabolism of citrate to fatty acids is likely reduced due to decreased ACLY (ATP citrate lyase), ACSL4 (Acetyl-CoA synthase 4), ACSL5 (Acetyl-CoA synthase 5), FASN (fatty acid synthase), and TECER. Since conversion directly to mitochondrial αKG is blocked by substantially decreased IDH2, 3A, 3B levels (Fig. 4c), while cytoplasmic IDH1 is largely unchanged, citrate is likely shunted cytoplasmically to αKG or glutamate and back into the TCA cycle to undergo another anaplerotic cycle. However, the exact route cannot be determined from these protein-level results. Fourth, while mitochondrial malate dehydrogenase (MDH2) is largely unchanged upon arginine starvation, MDH1 is upregulated by 1.6-fold, suggesting that OAA may be preferentially formed from malate that has been exported to the cytoplasm (Fig. 4c). Taken together, the results from the ABPP analysis suggest that glutamine-based production of OAA and aspartate is driven by three potential pathways: increased TCA cycle activity, anaplerotic carboxylation of pyruvate, and inhibition of lipid metabolism that recycles cytoplasmic citrate back to the TCA cycle.

### Arginine starvation induces adaptive kinomic changes driving MYC–MAX activation in SKLMS-1 cells

**Fig. 5:**
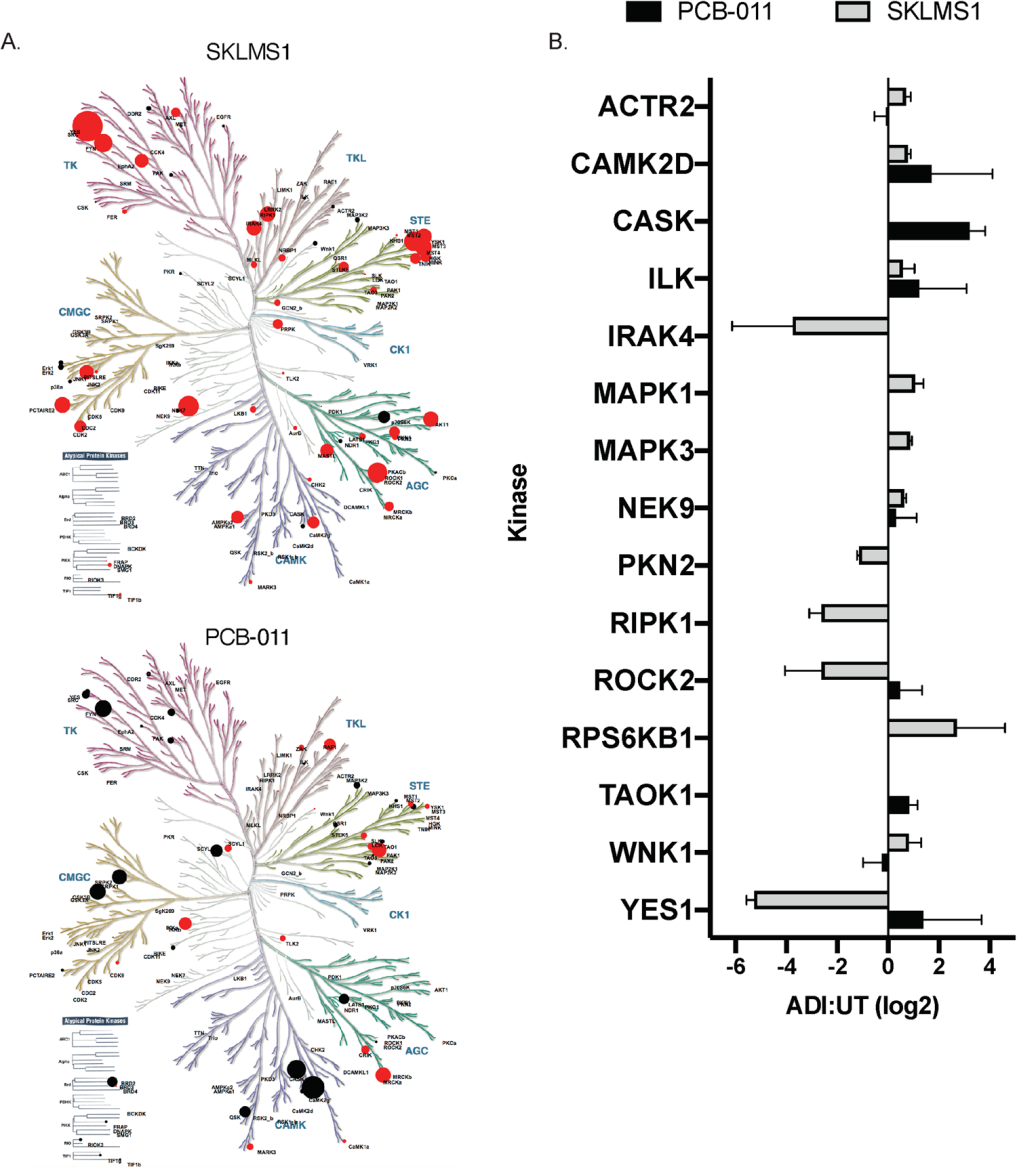
The adaptive kinome of SKLMS-1 and PCB-011 cells in response to ADI-PEG20-PEG20 includes ERK. **a** Kinases identified by ABPP are labeled. Size of the circle indicates relative changes in binding upon ADI-PEG20 treatment and is scaled from the log_2_ ratio. Color choices are assigned according to the directionality of deviation from no change (black, increased binding; red, decreased binding). Kinome tree illustration reproduced courtesy of Cell Signaling Technology, Inc. (http://www.cellsignaling.com). **b** Kinases detected by ABPP with a log_2_ fold change > |0.5 | . *N* = 3 biological replicates; data are represented as mean + SD.

The regulation of metabolic adaptation and reprogramming is highly pleiotropic, coordinating regulation of kinases, transcription factors, and other proteins across multiple signaling pathways^42^. Kinases are key transducers of signaling pathways, often clinically actionable, and can be unbiasedly profiled by ABPP^22,43^. Due to the fact that GSEA indicated that MAPKs were uniquely upregulated in SKLMS-1 cells upon arginine starvation (Fig. 4a, b), we further investigated the kinomic changes of SKLMS-1 and PCB-011 cells in response to ADI-PEG20. Consistent with phosphoproteome regulation (Fig. 3a, b), ADI-PEG20-sensitive SKLMS-1 cells have a much more dynamic response to ADI-PEG20 treatment than PCB-011, and the patterns of kinases that are regulated are distinct (Fig. 5a). Kinases with altered ABPP levels (*p* ≤ 0.05) are shown in Fig. 5b, which includes 14 kinases in SKLMS-1 cells compared to two in PCB-011 cells. Notably, while SKLMS-1 cells do not harbor activating mutations in ERK or AKT/mTOR signaling, ERK1 and ERK2 (MAPK3 and MAPK1, respectively) along with the ERK substrate p70 S6 kinase^21^ have the largest increases upon ADI-PEG20 treatment by ABPP profiling. Each of these kinases promote tumor growth and are capable of reprograming cellular metabolism^44,45^. Significantly, ERK activation has been implicated in the escape mechanism to ADI-PEG20 in melanoma^41^.

**Fig. 6:**
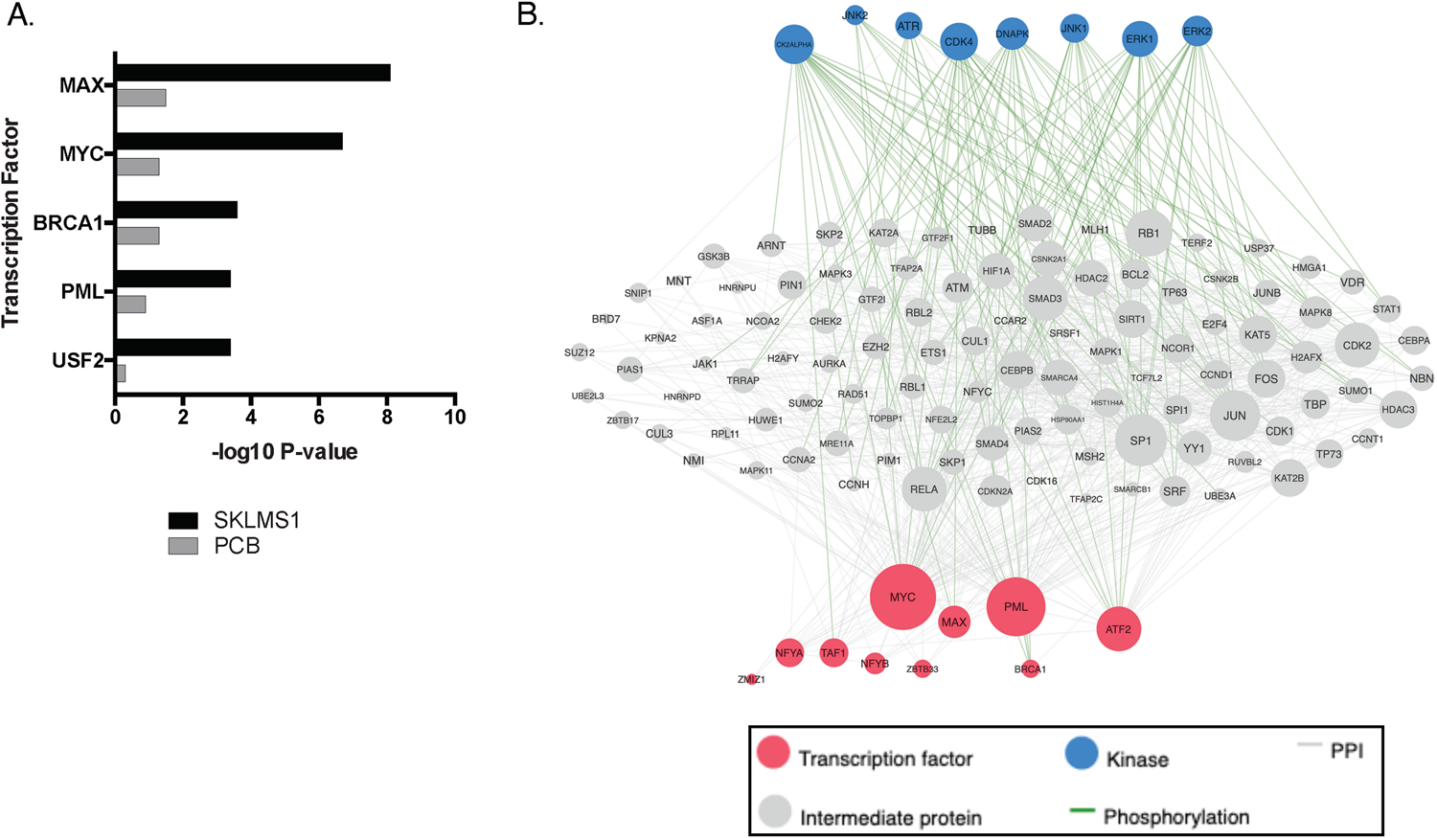
ADI treatment activates a signaling network driving Myc–Max activation. **a** X2K transcription factor enrichment analysis of SKLMS-1 and PCB-011 cells upon ADI treatment. *N* = 3 biological replicates. **b** X2K kinase network analysis based on ABPP profiling of SKLMS-1 cells upon ADI treatment.

In order to build a more complete picture of the regulatory networks involved in the response of SKLMS-1 and PCB-011 cells to arginine starvation, we performed X2K analysis on the ABPP data^37^. X2K incorporates kinomic and other changes in protein expression to infer regulatory networks. As X2K requires differentially expressed genes as input, all upregulated proteins with a nominally significant *p* value were included in the X2K analysis of each cell line. Myc, and its activating heterodimeric partner Max, were the two most overrepresented transcription factors in SKLMS-1 cells upon ADI-PEG20 treatment but were much less enriched in PCB-011 cells (Fig. 6a). Myc is stabilized in SKLMS-1 cells upon ADI-PEG20 treatment and the cMyc–Max heterodimerization inhibitor 10058-F4 blocks ADI-driven resistance consistent with this regulatory model^17^. Network analysis suggests that Myc and Max are driven by upstream activation of ERK1/2 (MAPK1/3, Fig. 6b). ERK can phosphorylate and stabilize Myc^41,46^, supporting this regulatory model upon arginine starvation in SKLMS-1 cells. Taken together, the ABPP profiling indicates adaptive changes in ERK1/2 upon ADI-PEG20 treatment and a concomitant activation of the cMyc–Max transcriptional network in ADI-PEG20-sensitive SKLMS-1 cells, but not in ADI-PEG20-resistant PCB-011 cells.

### Adapted resistance to ADI-PEG20 sensitizes cells to inhibition of the MEK-ERK-cMyc pathway

**Fig. 7:**
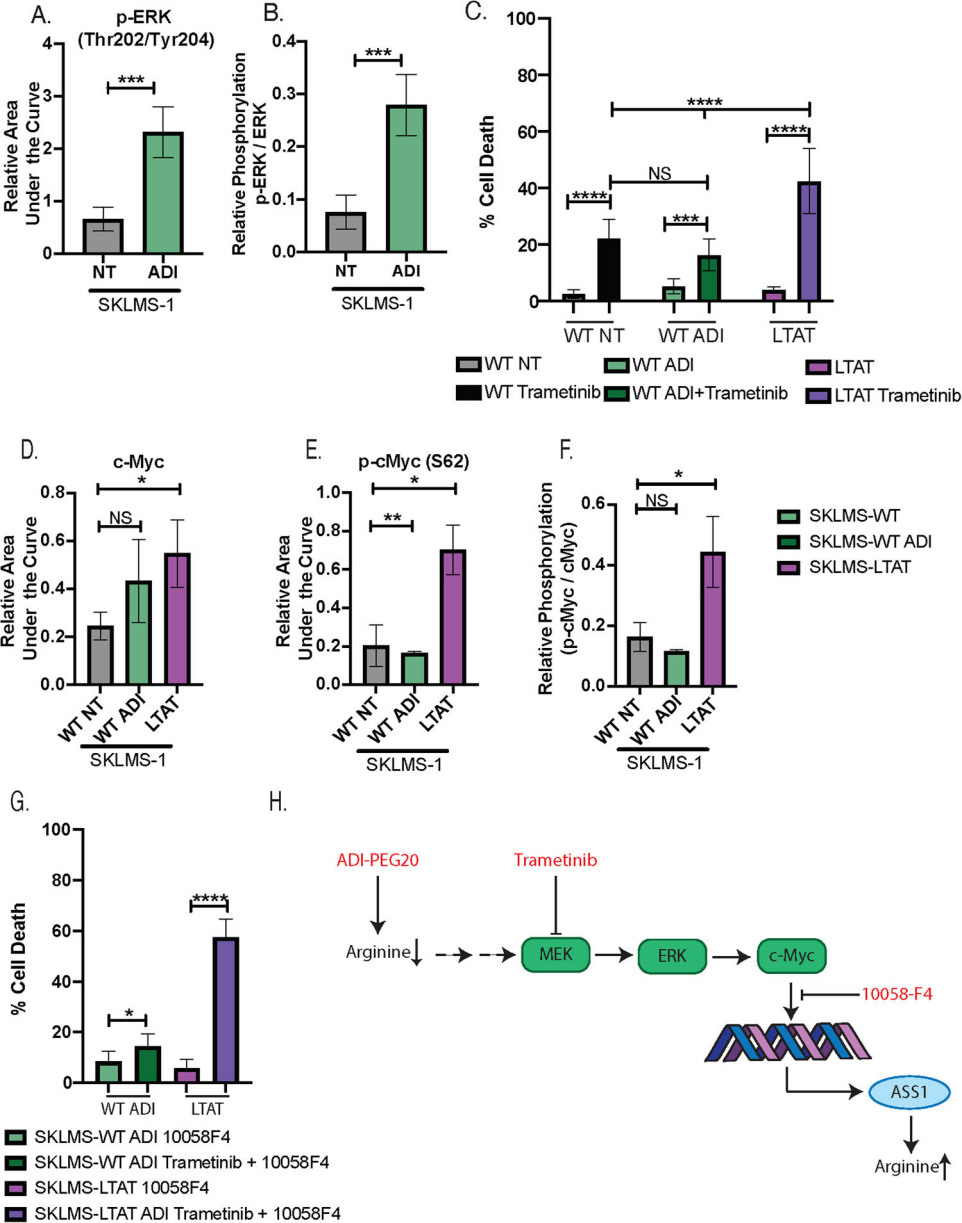
Adapted resistance to ADI-PEG20 sensitizes cells to inhibition of the MEK-ERK-cMyc pathway. **a** Protein expression of phospho-ERK (Thr202/Tyr204) compared in untreated SKMS-1 WT and ADI-treated SKLMS-1 WT at 72 h. Band density differences were plotted as phospho-ERK (Thr202/Tyr204) area under the curve normalized to total protein in the capillary (*N* = 3); data are represented as mean + SD. **b** Protein expression of phospho-ERK (Thr202/Tyr204) normalized to protein expression of ERK compared in untreated SKMS-1 WT and ADI-treated SKLMS-1 WT at 72 h. Data are represented as mean + SD (*N* = 3). **c** In vitro cell death response to Trametinib (1.6 µM) treatment in SKLMS-1 WT cell lines, SKLMS-1 WT ADI-PEG20 treated cell lines, and SKLMS-1 LTAT cell lines. Data are represented as mean ± SD (*n* = 3). NS *p* > 0.5, ***p < 0.001, ****p < 0.0001. **d** Protein expression of cMyc in untreated SKMS-1 WT, ADI-treated SKLMS-1 WT, and SLKMS-1 LTAT at 72 h. Band density differences were plotted as cMyc area under the curve normalized to total protein in the capillary (*n* = 3); data are represented as mean + SD. **e** Protein expression of phospho-cMyc (S62) in untreated SKMS-1 WT, ADI-treated SKLMS-1 WT, and SLKMS-1 LTAT at 72 h. Band density differences were plotted as phospho-cMyc (S62) area under the curve normalized to total protein in the capillary (*n* = 3); data are represented as mean + SD. **f** Protein expression of phospho-cMyc (S62) normalized to protein expression of cMyc compared in untreated SKMS-1 WT, ADI-treated SKLMS-1 WT, and SLKMS-1 LTAT at 72 h. Data are represented as mean + SD (*N* = 3). **g** In vitro cell death response to 1058-F4 (5 µM) treatment or combined 10058-F4 (5 µM) + Trametinib (1.6 µM) treatment in SKLMS-1 WT cell lines, SKLMS-1 WT ADI-PEG20-treated cell lines, and SKLMS-1 LTAT cell lines. Data are represented as mean ± SD (*N* = 3). NS *p* > 0.5, ***p < 0.001, ****p < 0.0001. **h** Schematic diagram of proposed model of ERK/cMyc mediated escape from ADI-PEG20 treatment.

In order to biochemically and functionally validate the activity of the ERK/cMyc pathway identified by proteomics, we first evaluated ERK expression and activating phosphorylation in SKLMS-1 cell lines treated with ADI-PEG20 for 72 h. Capillary western analysis demonstrated no significant changes in protein expression of ERK1/2 between WT and ADI treated SKLMS-1 cells (data not shown). Consistent with the proteomic studies, 72 h of ADI-PEG20 treatment resulted in a significant increase in activating phosphorylation of ERK (Thr202/Tyr204) relative to the untreated cells (Fig. 7b). Additionally, the ratio of phosphorylated ERK (Thr202/Tyr204) to unphosphorylated ERK was significantly increased in the ADI-PEGD20 treated SKLMS-1 cells (Fig. 7c).

Secondly, to investigate the functional significance of ERK activation in the escape response to ADI-PEG20, cell proliferation experiments were conducted using the Mek inhibitor Trametinib in ADI-PEG20-sensitive SKLMS-1 WT cells and ADI-PEG20-resistant SKLMS-1 LTAT (long-term ADI-PEG20 treated) cells^15^. Trametinib inhibits MEK-mediated ERK activation and prevents activation of its downstream signaling targets^47,48^. Therefore, Trametinib was utilized to interrogate dependency upon ERK signaling in the development of resistance to arginine starvation. SKLMS-1 WT cells halt cellular proliferation in the presence of ADI-PEG20, resulting in relative protection from cell death during the acute phase ADI-PEG20 resistance. Conversely, SKLMS-1 LTAT cells stably express ASS1, resulting in resistance and proliferation in the presence of ADI-PEG20. Therefore, SKLMS-1 WT ADI treated cell lines were utilized to evaluate acute response to ADI-PEG20 treatment and SKLMS-1 LTAT cell lines were utilized to evaluate adapted resistance^15^. Cell death in SKLMS-1 cell lines increased with Trametinib treatment (Fig. 7c). However, ADI-PEG20 resistant SKLMS-1 LTAT cell lines exhibit significantly higher rates of cell death with Trametinib than either SKLMS-1 WT untreated or SKLMS-1 WT ADI-PEG20 treated cells (Fig. 7c). These data indicate increased dependence upon ERK signaling in in the context of adaptive resistance to arginine starvation, supporting the proposed activation of ERK in the ADI-PEG20 escape response.

In addition to ERK upregulation, the X2K analysis indicated significant upregulation of the Myc–Max pathway in response to treatment with ADI-PEG20 in SKLMS-1 (Fig. 6a). Therefore, the functional role of cMyc in the development of ADI-PEG20 resistance was also evaluated. Capillary western analysis revealed modest increases in cMyc expression between SKLMS-1 WT untreated and SKLMS-1 WT ADI treated cells. While, ADI-PEG20 resistant SKLMS-1 LTAT cells exhibited a significant increase in cMyc protein expression relative to ADI-PEG20-sensitive SKLMS-1 WT cells (Fig. 7d), suggesting increased stabilization of cMyc as cells develop resistance to arginine starvation. Indeed, stabilizing phosphorylation (S62) and the ratio of phosphorylated to unphosphorylated cMyc was significantly increased the ADI-PEG20-resistent SKLMS-1 LTAT cells (Fig. 7e, f), supporting upregulation of this pathway in ADI-PEG20 escape^46^.

In order to evaluate the functional significance of cMyc in the putative escape pathway, a specific inhibitor of the Myc–Max interaction (10058-F4) was utilized in isolation and in combination with Trametinib. Treatment of ADI-PEG20 SKLMS-1 WT and LTAT cell lines with 10058F4 alone did not result in substantial cell death (Fig. 7g) However, co-treatment with Trametinib and 10058-F4 caused significant cell death in SLKMS-1 LTAT cell lines but only resulted in a modest increase in cell death in SKLMS-1 WT cell lines (Fig. 7g). These data suggest synergistic or additive effects of MEK inhibition and cMyc inhibition in the context of ADI-PEG20 resistance. Cumulatively, these data support the proteomic analysis and suggest activity of the ERK/cMyc signaling pathway in the escape mechanism of SKLMS-1 to ADI-PEG20.

## Discussion

Acquired resistance to anticancer therapy remains a major challenge and often occurs in the absence of genetic mutations. Many cancers are arginine auxotrophic due to silencing of ASS1 and/or argininosuccinate lyase^4^. These tumors are sensitive to ADI-PEG20, which converts arginine to citrulline^49^. However, monotherapy with ADI-PEG20 ultimately results in the development of tumor resistance through re-expression of ASS1, metabolic reprogramming^15^, and Myc stabilization^17^. As the cellular signaling pathways that mediate these changes are not completely understood, we focused on identifying the proteomic adaptations that facilitate metabolic reprogramming, and ultimately resistance to arginine deprivation in ADI-PEG20 sensitive cells.

In this study we employed phosphoproteomics and ABPP to characterize how the proteome of SKLMS-1 cells adapts upon arginine starvation. These methods proved to be complementary, and by integrating these data with existing metabolomics data^15^, we were able to gain unique insights into the regulatory networks involved in ADI-PEG20 resistance in ASS1-negative sarcoma. In line with existing literature, we identified adaptive kinomic changes in ADI-PEG20-sensitive SKLMS-1 cells, including upregulation of ERK1 and ERK2. Network analysis suggested that this ERK upregulation stimulates a Myc–Max transcriptional network (Figs. 4a and 6a). Myc in this context has been demonstrated to promote re-expression of ASS1^41^. In addition, Myc is able to promote glutamine anaplerosis^50^, but in the setting of arginine deprivation it is not known how proteomic changes facilitate this metabolic reprogramming^51–53^. The cellular signaling events that promote reprogramming of glutamine metabolism in this context are the subject of ongoing research. We find that regulation of multiple proteins likely contributes to increased flux from glutamine to OAA (glutamine anaplerosis), direct oxaloacetate production from pyruvate by increasing PC (pyruvate anaplerosis), and upregulation of citrate synthase combined with inhibition of lipid synthesis to recycle citrate for TCA anaplerosis (Fig. 4b, c). Furthermore, consistent with the proteomic analysis, we have identified a novel sensitivity in vitro to inhibition of ERK activation with trametinib in the context of ADI-PEG20 adapted resistance.

The reprogramming of cancer metabolism is a critical factor promoting tumorigenesis and drug resistance. Proteomic regulation is essential to metabolic reprogramming^54^, such as PKM2 tetramerization that promotes the Warburg effect^55^. While the limited number of cellular metabolites^56^ make metabolomic profiling relatively routine, the vast complexity of proteomic regulation remains challenging and the myriad of potential mechanisms by which proteomic changes drive metabolic reprogramming remain incompletely understood. The combination of multiple proteomic and metabolomic techniques will be essential for resolving these complex pathways, as highlighted by this study. The precision of ABPP is limited by allosteric modulation by ATP, protein-protein interactions, and other factors; therefore, the sole use of ABPP for the elucidation of complex metabolic networks is unlikely to provide a comprehensive pathway analysis. However, combining ABPP with other -omics, such as phosphoproteomics and metabolomics, allows for a more complete understanding of these interconnected systems. Systems level analysis pairing global, unbiased, and integrative proteomic and metabolic analyses have recently been performed in models of plants^57–59^, parasites, and antibiotic resistance^60,61^. However, utilization of integrative proteomic–metabolomic analysis has been limited with respect to modeling drug resistance and metabolism in cancer models^62–65^. Our exploration of ADI-PEG20 resistance elucidates numerous changes consistent with metabolomics and finds that these two -omics approaches provide complementary insight.

A full understanding of the metabolic and proteomic adaptation to ADI-PEG20 is needed for arginine starvation to become a mainstay of cancer treatment. The metabolic changes that promote escape from arginine starvation induce a new transcriptional profile as well as significant alterations in protein regulation and activity. The changes that facilitate escape from arginine deprivation also limit the metabolic flexibility of cells. It has yet to be determined if these metabolic adaptations are permanent choices, but as long as the stress of extracellular arginine starvation is present, the ability of a tumor to return to its baseline metabolism is limited. Understanding the metabolic changes that occur upon treatment with metabolically targeted compounds in concert with associated proteomic changes provides insight into cellular resistance mechanisms and may inform the development of more efficient multiagent therapies.
